# Role of Coronary Myogenic Response in Pressure-Flow Autoregulation in Swine: A Meta-Analysis With Coronary Flow Modeling

**DOI:** 10.3389/fphys.2018.00580

**Published:** 2018-05-23

**Authors:** Gregory M. Dick, Ravi Namani, Bhavesh Patel, Ghassan S. Kassab

**Affiliations:** California Medical Innovations Institute, San Diego, CA, United States

**Keywords:** arteriole, microcirculation, smooth muscle, myography, coronary blood flow

## Abstract

Myogenic responses (pressure-dependent contractions) of coronary arterioles play a role in autoregulation (relatively constant flow vs. pressure). Publications on myogenic reactivity in swine coronaries vary in caliber, analysis, and degree of responsiveness. Further, data on myogenic responses and autoregulation in swine have not been completely compiled, compared, and modeled. Thus, it has been difficult to understand these physiological phenomena. Our purpose was to: (a) analyze myogenic data with standard criteria; (b) assign results to diameter categories defined by morphometry; and (c) use our novel multiscale flow model to determine the extent to which *ex vivo* myogenic reactivity can explain autoregulation *in vivo*. When myogenic responses from the literature are an input for our model, the predicted coronary autoregulation approaches *in vivo* observations. More complete and appropriate data are now available to investigate the regulation of coronary blood flow in swine, a highly relevant model for human physiology and disease.

## Introduction

Myogenic reactivity can be described as the mechanism underlying the Bayliss effect (Bayliss, 1902). That is, when blood pressure is elevated, arteries distend, and the smooth muscle cells in the vascular wall respond by contracting. Autoregulation is the phenomenon where coronary blood flow remains relatively constant over a wide range of perfusion pressures (Mosher et al., 1964). The Hagen-Poiseuille relationship predicts that—in the absence of other changes—when the pressure gradient increases, flow should increase. This is because flow is directly related to the pressure gradient and to the 4th power of the vessel radius, while inversely related to blood viscosity and vessel length. Thus, one reasonable assumption to explain this autoregulatory behavior is that vessels of the coronary tree actively adjust their diameter as pressure is varied. The mechanism by which coronary resistance vessels alter their diameter in response to pressure changes is the myogenic response. We aim to synthesize the relevant existing data for coronary myogenic responses and autoregulation in a single species: swine. There are, of course, many studies from other species and they are extremely important because of the mechanistic insights provided. One of the most complete data sets is available from swine, however, and these animals are invaluable experimental models because of similarities with humans in coronary anatomy, physiology, and disease (Suzuki et al., 2011; Lelovas et al., 2014).

### Myogenic responses

**Figure 1:**
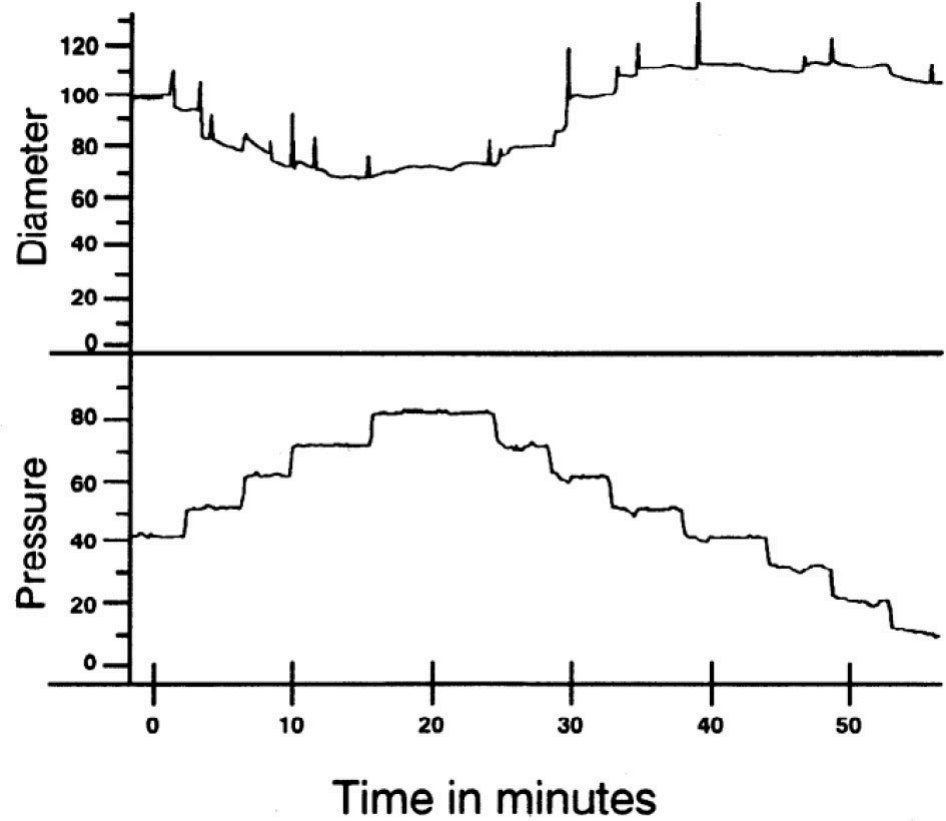
Example of myogenic reactivity in a swine coronary arteriole. Muller et al. demonstrated how coronary arteriolar diameter (in μm; **top**) changed as transmural pressure (in mmHg; **bottom**) was varied (Muller et al., 1993; reproduced with permission). As distending pressure was increased from 40 to 80 mmHg in 10 mmHg increments, the steady-state diameter decreased. When transmural pressure was reduced from 80 mmHg, diameter increased.

The myogenic response is generally thought of as vasoconstriction in response to increased intraluminal pressure, but reducing pressure also elicits vasodilation (Figure 1). The myogenic response is typically studied *ex vivo* using pressure myography methods. Small arteries and arterioles are dissected from living tissue, bathed in physiological solutions at body temperature, cannulated, connected to a pressure source, and imaged to determine the inner diameter as the distending internal pressure is varied with no flow. The vascular myogenic response and its mechanisms have been the subject of many studies and reviews (Davis, 2012; Hill and Meininger, 2012). From a teleological perspective, myogenic responses may represent the efforts of a blood vessel to minimize the stress on its wall. This is because, according to the law of Laplace, mean wall stress is directly proportional to the product of pressure and radius, while inversely related to wall thickness. Thus, if blood pressure were to increase, elevated vascular wall tension could be mitigated by an arteriole actively decreasing its radius and/or thickening its wall. Further, myogenic responses could provide a certain degree of constriction at normal intraluminal pressures (i.e., give the vessel a basal, intrinsic, or spontaneous tone from which to deviate). This would allow coronary vascular diameter, and thus resistance, to change in either direction through the action of vasodilator and vasoconstrictor influences such as metabolic demands, neural activity, and paracrine stimuli (Duncker and Bache, 2008; Tune, 2014; Goodwill et al., 2017). This idea of intrinsic tone in a coronary arteriole is an important one, because flow is related to diameter in a power-law manner. Thus, very small adjustments in coronary arteriolar diameter in either direction have substantial effects on myocardial blood flow.

**Figure 2:**
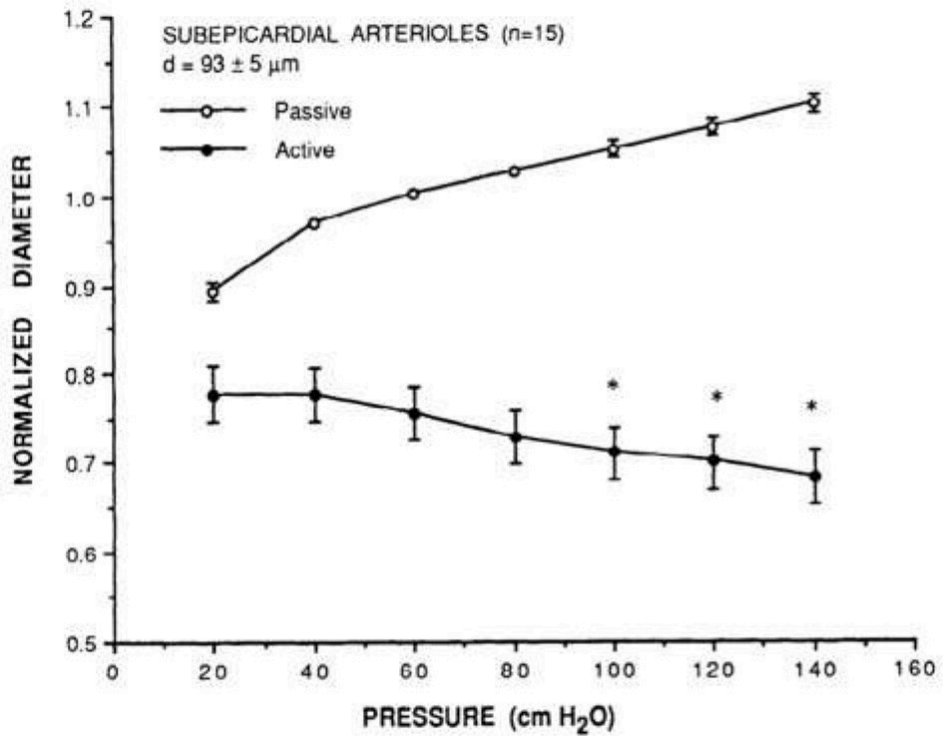
Prototypical description of coronary myogenic reactivity. Kuo et al. showed the pressure-diameter relationship of swine coronary arterioles (Kuo et al., 1988; reprinted with permission). The active curve was observed under control conditions, while the passive curve was measured in the presence of 100 μM sodium nitroprusside, a source of the vasodilator nitric oxide. Diameters are normalized to the passive diameter at 60 cmH_2_O (44.1 mmHg). Asterisks indicate an active diameter significantly different from that at 60 cmH_2_O.

The first study of coronary myogenic reactivity in swine (or any species, for that matter) was published in 1988, demonstrating what has come to be considered classic coronary myogenic responsiveness (Kuo et al., 1988; Figure 2). The PubMed engine was used to search the MEDLINE database for published studies focusing on myogenic responses in swine coronary small arteries and arterioles. Using the search terms swine, coronary, and myogenic returned 54 publications. A total of 11 relevant studies are identified in Table 1. Between 1988 and 1991, Kuo et al. published three seminal papers describing fundamental properties of the myogenic response in swine coronary arterioles. First, the myogenic responsiveness of subepicardial arterioles exceeded that of similarly sized subendocardial arterioles; i.e., a transmural gradient of myogenic reactivity exists in the swine heart (Kuo et al., 1988). Second, myogenic responses were similar in swine coronary arterioles with and without functional endothelium, indicating that the behavior is inherent to the smooth muscle (Kuo et al., 1990a). Third, pressure (causing myogenic vasoconstriction) and flow (producing endothelium-dependent vasodilation; Kuo et al., 1990b) interact to determine the resulting vascular tone in swine coronary arterioles with intact endothelium (Kuo et al., 1991). In the ensuing years, several other groups published studies documenting how the myogenic responses of swine coronary arterioles were impacted by exercise, clinical interventions, or cardiovascular disease. For instance, Muller et al. demonstrated that endurance exercise training increased the myogenic reactivity of coronary arterioles from swine (Muller et al., 1993), while Sellke and colleagues documented the deleterious effects of coronary bypass and cardioplegia on the myogenic reactivity of swine coronary arterioles (Wang et al., 1995). Most recently, Sorop et al. demonstrated that myogenic responses were blunted downstream of a chronic coronary occlusion in swine (Sorop et al., 2008).

**Table 1:** Characteristics of swine and their arterioles in 11 previous studies of coronary myogenic reactivity.

**References**	**Variety**	**Gender**	**Weight (kg)**	**Age (mo.)^b^**	**Layer^c^**	**Territory^d^**	**Diameter^e^**
Kuo et al., 1988	Domestic	M, F	10	1-2	Epi and Endo	LAD and LCx	134 and 136^f^
Kuo et al., 1990a	Domestic	M, F	10^a^	1-2	Epi	LAD and LCx	91^f^
Kuo et al., 1991	Domestic	M, F	11-22^a^	1.5-2.5	Epi	LAD and LCx	85^f^
Muller et al., 1993	Yucatan	F	25-40	6^+^	Epi	LV wall	124-129
Rajagopalan et al., 1995	Domestic	M, F	10	1-2	Epi	LAD	188
Wang et al., 1995	Domestic	M, F	19-23	2-3	Epi	LCx	150
Wang et al., 1997	Domestic	M, F	20-25	2-3	Epi	LAD	168
Tofukuji et al., 1997a	Domestic	M, F	20-25	2-3	Epi	LAD	141
Tofukuji et al., 1997b	Domestic	M, F	20-25	2-3	Epi	LAD	138
Liao and Kuo, 1997	Domestic	M, F	16-30^a^	2-3	Epi	LAD and LCx	254, 164, 99, and 64^f^
Sorop et al., 2008	Domestic	M, F	66	4^+^	Endo	LAD and LCx	229

a Weight estimated from growth charts using age provided.

b Approximate age estimated from weight using growth chart. Plus sign (+) signifies that age may be greater than indicated number of months.

c Indicates whether vessels were from subepicardium (Epi) or subendocardium (Endo).

d Left anterior descending (LAD) artery, left circumflex (LCx) artery, left ventricular (LV) wall.

e Passive inner diameter @ 80 mmHg (μm).

f Average of passive diameters at 73.5 and 88.2 mmHg.

### Coronary autoregulation

**Figure 3:**
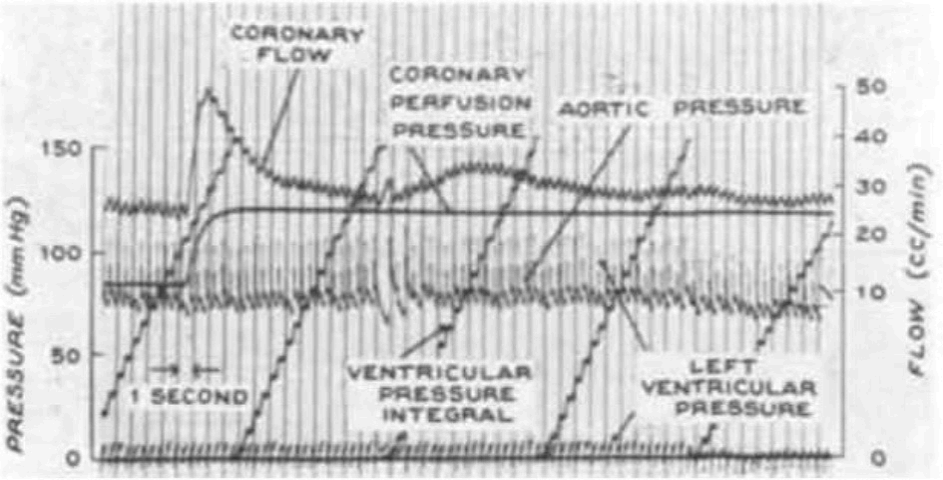
A representative tracing of coronary pressure-flow autoregulation in a dog from the classic study of Mosher et al. (1964; reproduced with permission). Note that as coronary perfusion pressure is suddenly increased from 82 to 110 mmHg, coronary blood flow transiently increases, but then rapidly returns toward its previous level.

**Figure 4:**
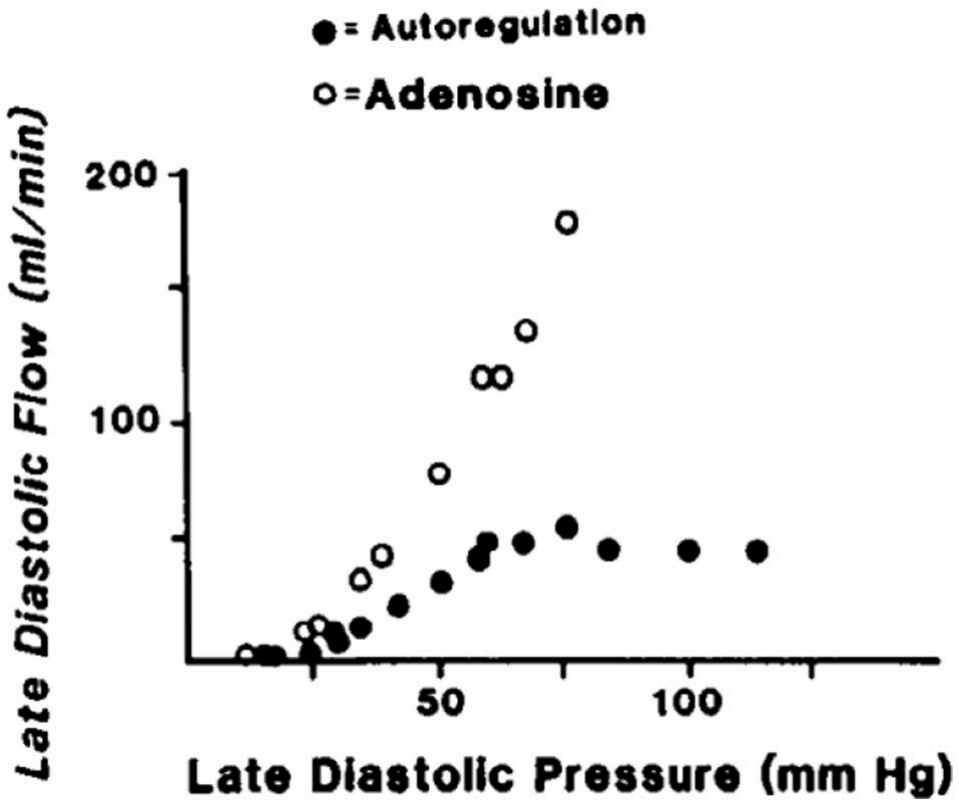
An example of coronary pressure-flow autoregulation in swine (Pantely et al., 1985; reprinted with permission). Flow in the LAD artery was measured in an open-chest, anesthetized pig. An inflatable perivascular occluder was used to constrict the artery while pressure and flow distal to the occlusion was measured. This was done before (autoregulation) and after intracoronary infusion of the vasodilator adenosine.

Given a constant myocardial oxygen demand, perfusion can remain relatively constant over a considerable pressure range (Mosher et al., 1964). One idea is that this coronary autoregulation may be mediated, at least in part, through pressure-induced changes in the diameter of coronary vessels (Johnson, 1980, 1986; Hoffman and Spaan, 1990). In other words, coronary vascular resistance changes as pressure is varied to maintain a relatively constant myocardial blood flow. An example of coronary pressure-flow autoregulation is shown in Figure 3. PubMed was used to search the MEDLINE database to find studies that focused on coronary pressure-flow autoregulation in swine. Many references (>300 each) were returned when performing searches with the terms porcine, coronary, and autoregulation or swine, coronary, and autoregulation. Ten pertinent pressure-flow autoregulation studies were identified (Table 2). Some show the control (active or autoregulated) response while others show the passive (or maximally dilated) response. A few studies show both behaviors. An example of coronary pressure-flow autoregulation in swine is shown in Figure 4.

**Table 2:** Studies of coronary pressure-flow relationships in swine.

**References**	**Variety**	**Gender**	**Weight (kg)**	**Age (mo.)^a^**	**Territory^b^**	**Autoregulated?**	**Dilated?**
Pantely et al., 1985	Domestic	NS	29-55	3-6	LAD	Yes	Yes
Johnson et al., 1988	Domestic	M, F	26-45	2.5-4	LCx	Yes	Yes
Schulz et al., 1991	Domestic	NS	20-45	2.5-4	LAD	Yes	No
McFalls et al., 1991	Domestic	M, F	24-42	2.5-4	LAD	Yes	Yes
Chilian, 1991	Domestic	M, F	7-15	1-2	LAD and LCx	No	Yes
Guth et al., 1991	Göttingen	M, F	25-35	3-6	RCA^c^	Yes	No
Duncker et al., 1992	Domestic	M, F	25-45	2.5-4	LAD	No	Yes
Shnier et al., 1994	Domestic	NS	40-50	5-6	LAD	Yes	No
Berwick et al., 2012	Ossabaw	NS	30-60	3-6	LAD	Yes	No
Schampaert et al., 2013	Domestic	NS	NS	NS	LAD and LCx^d^	No	Yes

NS, Not specified.

a Approximate age estimated from weight using growth chart.

b Left anterior descending (LAD), left circumflex (LCx), and right coronary (RCA) artery.

c In addition to the right ventricle, the RCA perfuses the interventricular septum. Only the septal data were included for analysis here.

d Total coronary blood flow was multiplied by 68.3% to estimate left ventricular perfusion (Feigl et al., 1990).

Whether myogenic responses play a role in coronary pressure-flow autoregulation was debated in the past (Dole, 1987; Feigl, 1989). This debate centered on three points: (a) the myogenic response of isolated coronary arterioles had not yet been observed; (b) indirect assessments of myogenic behavior (e.g., hyperemic responses following brief coronary occlusions) were equivocal due to the overriding effects of metabolism; and (c) there had not been a direct assessment of coronary myogenic behavior *in vivo* (e.g., with intravital microscopy in the beating heart). Most of these issues have been addressed, as the myogenic responses of isolated coronary are now widely recognized and intravital microscopy studies of arterioles *in vivo* have been completed. Intravital microscopy shows that coronary arterioles dilate as pressure is reduced (Chilian and Layne, 1990; Kanatsuka et al., 1990; Merkus et al., 2001), but experiments with increased pressures are lacking. The scarcity of data with increasing pressure is likely because it is more practical to reduce coronary pressure without altering myocardial oxygen demand. It should be recognized that there are other mechanisms which contribute to coronary pressure-flow autoregulation (e.g., by metabolic and endothelial influences), but coronary myogenic responses are widely believed to be fundamental to the phenomenon.

Three groups have successfully modeled coronary pressure-flow autoregulation by including a myogenic mechanism. Liao and Kuo (1997) generated a model that qualitatively reproduced the coronary pressure-flow relationship observed in Langendorff-perfused hearts (Ueeda et al., 1992). The model of Cornelissen and colleagues incorporated a network of vessels with diameter-dependent myogenic responses and generated theoretical pressure-flow curves with prominent autoregulation (Cornelissen et al., 2000, 2002). Most recently, Namani et al. provided an integrative model of coronary flow based on a realistic anatomy, active and passive flow determinants, and myogenic reactivity data (Namani et al., 2018). While important mechanistic insights were provided by these studies, a limitation of the previous modeling efforts is that they relied upon data from dissimilar species and/or *ex vivo* active autoregulation data (i.e., isolated hearts in which coronary flow typically exceeds values seen *in vivo*). All modeling studies were informed by myogenic responses from swine coronary arterioles, but none considered the coronary pressure-flow relationship in swine (Table 2; Figure 4).

Eliminating as many potential species- and method-related discrepancies from the input data sets for coronary myogenic responses and pressure-flow autoregulation may improve model output. Our meta-analysis has the following three goals. First, we analyzed previous studies of swine coronary myogenic responses with standard criteria. Particularly, we aimed to simplify inter-study comparisons by converting all units (to μm and mmHg) and applying a single method of presentation and analysis. Second, we assigned results to diameter categories defined by the morphometry of Kassab et al. (1993). This should facilitate comparisons between studies, as myogenic behavior is reported to be diameter-dependent (Liao and Kuo, 1997). Third, we compiled studies of coronary pressure-flow autoregulation from swine, then used myogenic responsiveness in porcine coronary arterioles to compute the pressure-flow autoregulation profile and compare it to what has been observed in the same species.

## Collecting and analyzing existing data

It was necessary to extract data from original reports (Tables 1, 2) for our analysis. This was achieved by obtaining Portable Document Files and analyzing digital images of the figures with WebPlotDigitizer (https://automeris.io/WebPlotDigitizer by Ankit Rohatgi, Austin, TX). Arteries and arterioles of different calibers were assigned to specific categories in a modified Strahler scheme based on morphometric data from the swine coronary circulation provided by Kassab et al. (1993). In this anatomical framework, the capillary is considered order 0. Upstream vessels are numbered sequentially. For the left anterior descending (LAD) artery perfusion territory, arterial segments range from 9.2 μm (order 1; the precapillary arteriole) to 3.2 mm (order 11; at the origin) (Kassab et al., 1993). In the myocardial region supplied by the left circumflex (LCx) artery, there are 10 arterial branch orders upstream of the capillary ranging from 9.2 μm to 2.6 mm (Kassab et al., 1993). Branches were assigned to orders based on their passive inner diameter at 80 mmHg; therefore, data from the studies of myogenic reactivity were sorted according to the same characteristic. Diameter category boundaries (rounded to the nearest 0.1 μm with no overlap) were using Equations (1, 2):

(1) $$\begin{array}{r} {\text{D}}_{\text{min}}=\left({\text{D}}_{\left(\text{n}\right)}-\text{S}{\text{D}}_{\left(\text{n}\right)}+{\text{D}}_{\left(\text{n}-1\right)}+\text{S}{\text{D}}_{\left(\text{n}-1\right)}\right)/2 \end{array}$$

(2) $$\begin{array}{r} {\text{D}}_{\text{max}}=\left({\text{D}}_{\left(\text{n}+1\right)}-\text{S}{\text{D}}_{\left(\text{n}+1\right)}+{\text{D}}_{\left(\text{n}\right)}+\text{S}{\text{D}}_{\left(\text{n}\right)}\right)/2 \end{array}$$

**Figure 5:**
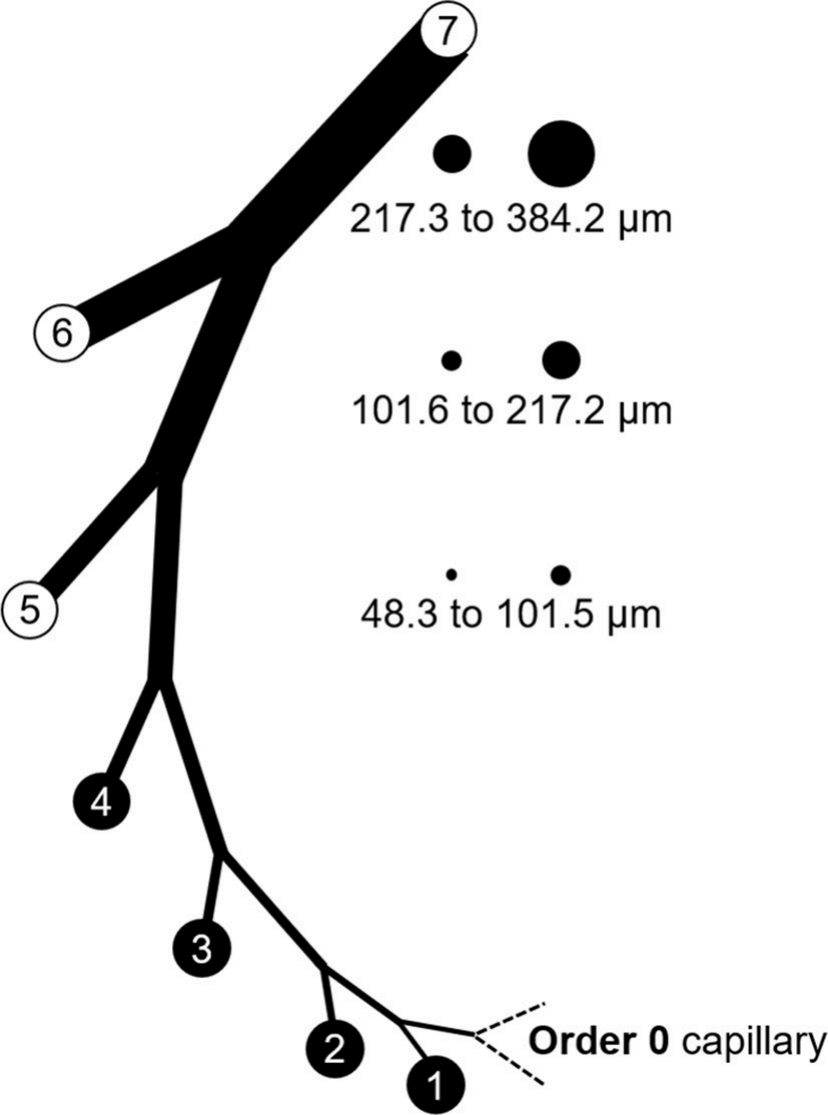
Branch order patterns and availability of myogenic reactivity data from swine. The cartoon shows branches from the capillary (order 0) to order 7. The circles and numbers to the right represent the relative sizes and exact diameters of vessels in orders 5, 6, and 7 of the LAD perfusion territory. Equivalent diameter ranges for those same orders in the LCx territory would be 52.1 to 101.7, 101.8 to 202.6, and 202.7 to 363.8 μm. Published data for myogenic reactivity in swine coronary arterioles are available for orders 5–7.

D_min_ and D_max_ are minimum and maximum diameters for a category, D is diameter, SD is the standard deviation, and (n) represents an order with its downstream (n – 1) and upstream (n + 1) neighbors. The order numbering scheme is shown in Figure 5.

The passive vessel radius (*R*_*p*_) is a sigmoidal function of the intraluminal pressure (Δ*P*; Equation 3; Young et al., 2012).

(3) $$\begin{array}{r} R_{p}\left(\Delta P\right)=B_{p}+\frac{A_{p}-B_{p}}{\pi }\left[\frac{\pi }{2}+arctan\left(\frac{\Delta P-\phi_{p}}{C_{p}}\right)\right] \end{array}$$

*A*_*p*_ and *B*_*p*_ are the maximum and minimum vessel radii, ϕ_*p*_, is the pressure corresponding to the mean vessel radius, and *C*_*p*_ is the pressure bandwidth for the transition in radius from *A*_*p*_ to *B*_*p*_. Radius in the active myogenic response (*R*_*m*_) is also a sigmoidal function of the intraluminal pressure (Equation 4).

(4) $$\begin{array}{r} \Delta R_{m}\left(\Delta \bar{P}\right)=\frac{\rho_{m}}{\pi }\left[\frac{\pi }{2}-\arctan\left({\left[\frac{\Delta \bar{P}-\phi_{m}}{C_{m}}\right]}^{2m}\right)\right] \end{array}$$

The four model parameters are: (1) the maximum decrease in vessel radius (or the peak amplitude), ρ_*m*_; (2) ϕ_*m*_ is the transvascular pressure at which the vessel radius decreases by ρ_*m*_ (the pressure at peak amplitude); (3) the pressure bandwidth of the vessel radius change is *C*_*m*_ (Namani et al., 2018); (4) and the exponent, *m*, is assumed to be 2.0 (Young et al., 2012).

The literature (Table 1) provides pressure-diameter relationships for swine coronary arterioles for vessel orders 5–7 only. This is likely for technical reasons, as the tiny arterioles of order 4 (< 48.3 μm) and below are challenging to cannulate and it would be difficult to image the lumen of the thicker walled vessels of order 8 (>384.2 μm) and above. To model flow control in the entire coronary tree, however, active constitutive properties are needed for vessels above and below orders 5–7. Thus, some assumptions and simplifications were introduced. Based on the weak or absent myogenic responses in vessels above order 7 (Nakayama et al., 1988; Liao and Kuo, 1997), these vessels were considered to have only passive properties in the model. Because capillaries (order 0) lack smooth muscle, these vessels were also considered to have only passive responses. Myogenic parameters for vessel orders 1–4 were extrapolated from the extracted myogenic parameters of vessels order 5–7. The longitudinal distribution of the myogenic parameters was fit with a three-parameter Weibull distribution function (Equation 5).

(5) $$\begin{array}{r} \left[\begin{array}{c} \rho_{m}\\ \phi_{m}\\ C_{m} \end{array}\right]=\left\{C{\left(\frac{R}{a}\right)}^{b-1}e^{-{\left(\frac{R}{a}\right)}^{b}}\right\} \end{array}$$

The Weibull distribution defines the myogenic response as a function of the vessel cast radius and serves as an input to the flow analysis in the coronary tree (Namani et al., 2018). Subepicardial and subendocardial vessels of the same order may have different myogenic responses (Kuo et al., 1988), which could affect the longitudinal distribution of myogenic parameters transmurally. The available data are predominantly from subepicardial vessels, whereas only two data sets are available for subendocardial vessels; therefore, there will be greater uncertainty in myogenic properties of vessels from this region.

To understand the effect of the myogenic response on coronary pressure-flow autoregulation, the flow regulation model was simulated with and without active myogenic responses in trees from the subepicardium and subendocardium. Trees were composed of 400 vessels to minimize computational effort. When active myogenic responses were removed, the vessels were given a basal tone (i.e., a degree of constriction that is independent of the transvascular pressure). The tone described here is meant to be of the same nature as myogenic contractions. That is, the tone is inherent to the smooth muscle itself (i.e., it is myogenic and not due to extrinsic factors), but does not vary with pressure. The prescribed basal tone (15%; an indicator of viable arterioles in *ex vivo* experiments Muller et al., 1993) was made uniform in all vessel orders (1–7) of the subtree to simplify the simulation.

## Compiling and integrating the existing data

**Figure 6:**
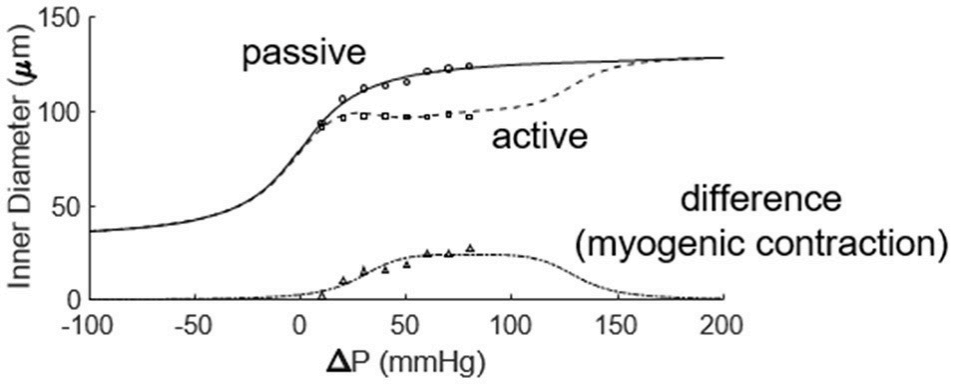
Fitting the active and passive pressure-diameter relationships with Equations (3, 4). The data were obtained from the study of Muller et al. (1993).

**Figure 7:**
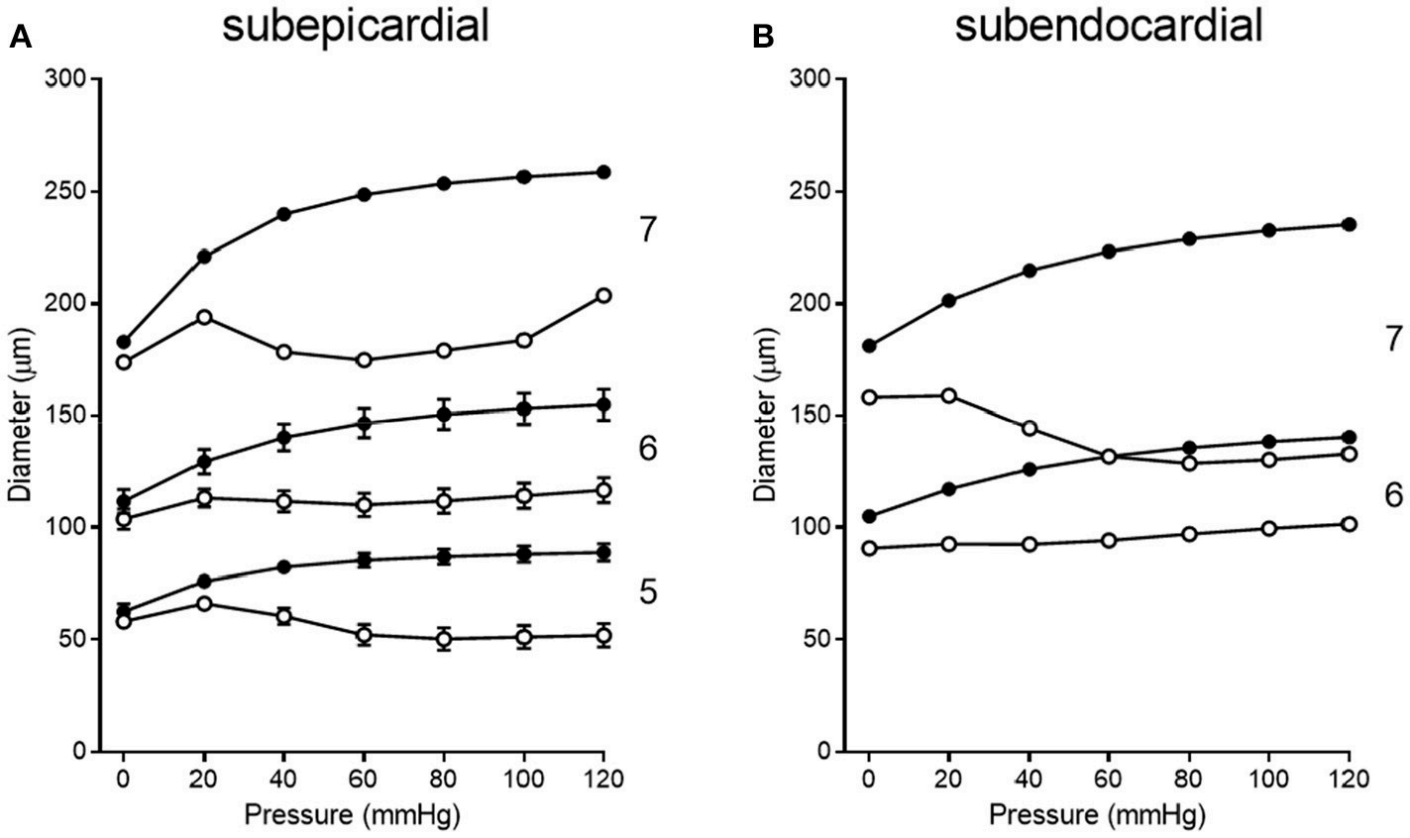
Composite pressure-diameter relationships for all studies of swine coronary arterioles listed in Table 1. Filled symbols represent the passive curves, while open symbols represent the active myogenic responses. Order numbers are given to the right of each data set. In the subepicardium **(A)**, data are available from order 7 (1 study), order 6 (9 studies), and order 5 (3 studies). For subendocardial vessels **(B)**, data are available for orders 7 and 6 (1 study each).

Pressure-diameter data from the 11 previous studies cover orders 5, 6, and 7. Data from subepicardial vessels span all three orders, while data from subendocardial vessels are available for only orders 6 and 7. There is some variability in the pressure ranges and units (e.g., cmH_2_O vs. mmHg) used to describe the results in the studies of Table 1. Further, pressure-diameter relationships from those studies are expressed differently (e.g., a percentage of the maximum diameter vs. μm). Thus, the data were extracted, converted to standard units of μm vs. mmHg, and assigned to the appropriate branch order and myocardial layer. Then those data were fit with Equations (3) (passive curve) and (4) (active myogenic response). Data are not available for the diameters of coronary arterioles at pressures greater than 100 mmHg, but data for coronary autoregulation extend past that pressure; therefore, pressure-diameter curves were extrapolated using the following logic. First, pressure-diameter data at higher pressures are available from mesenteric and femoral arterioles and can be used as a guide (Carlson and Secomb, 2005). These data show that the myogenic diameter converges with the passive vessel diameter at high transvascular pressures (100–200 mmHg). Second, Young et al. found that extrapolation of the myogenic pressure-diameter relationship beyond 100 mmHg is reasonable (Young et al., 2012). Third, Hamza et al. measured the passive pressure-diameter relationship of larger coronary vessels up to 150 mmHg and found a typical sigmoidal shape (Hamza et al., 2003). It is important to point out, however, that there are no data available to indicate whether the pressure-diameter relationships of isolated arterioles are reflective of *in situ* properties. Thus, our assumptions may need to be revisited. Example curve fits are shown in Figure 6. These curve fits were sampled in 20 mmHg increments from 0 to 120 mmHg to obtain data suitable for calculating mean (with standard error, where possible) pressure-diameter relationships in each available vessel order of the subepicardium and subendocardium (Figure 7). These pressure-diameter relationships are referred to as the “composite,” as they represent the average of responses available from the literature.

Our literature search identified 10 studies of coronary pressure-flow autoregulation in swine (Table 2). Eight were *in vivo* studies (Pantely et al., 1985; Johnson et al., 1988; Guth et al., 1991; McFalls et al., 1991; Schulz et al., 1991; Duncker et al., 1992; Shnier et al., 1994; Berwick et al., 2012), while two were *ex vivo* studies of isolated, blood-perfused swine hearts (Chilian, 1991; Schampaert et al., 2013). There were seven studies that provided active autoregulatory data (all of those were *in vivo* studies; see Table 2 for “Yes” in the “Autoregulated” column). Four of the eight *in vivo* studies provided pressure-flow data from vasodilated hearts (passive responses; see Table 2 for “Yes” in the “Dilated” column). Both *ex vivo* studies were sources of data for the pressure-flow relationship in the vasodilated (passive) coronary circulation only. Data were extracted from the studies, flows converted to ml/min/g (where necessary), and curve fitted. To determine flow per gram of myocardium, we estimated heart weight from body weight. In swine, the heart weight to body weight ratio is the same as humans (5 g/kg; Lelovas et al., 2014). To determine the weight of a particular perfusion territory (e.g., LCx or LAD), data from canine hearts were used (Feigl et al., 1990), as no similar data exist for swine. Feigl's analysis indicates that the LCx perfusion area is 39.0% of heart weight, while that of the LAD zone is 29.3%. For the active (autoregulated) response, data were fit with a third order polynomial (cubic; Equation 6) and the goodness of fit had R^2^ values between 0.94 and 0.99.

(6) $$\begin{array}{r} \text{f}\left(\text{x}\right)=\text{a}{\text{x}}^{3}+\text{b}{\text{x}}^{2}+\text{cx}+\text{d} \end{array}$$

For the passive (vasodilated) response, data were fit with a second order polynomial (quadratic; Equation 7) and the goodness of fit had R^2^ values above 0.98.

(7) $$\begin{array}{r} \text{f}\left(\text{x}\right)=\text{a}{\text{x}}^{2}+\text{bx}+\text{c} \end{array}$$

**Figure 8:**
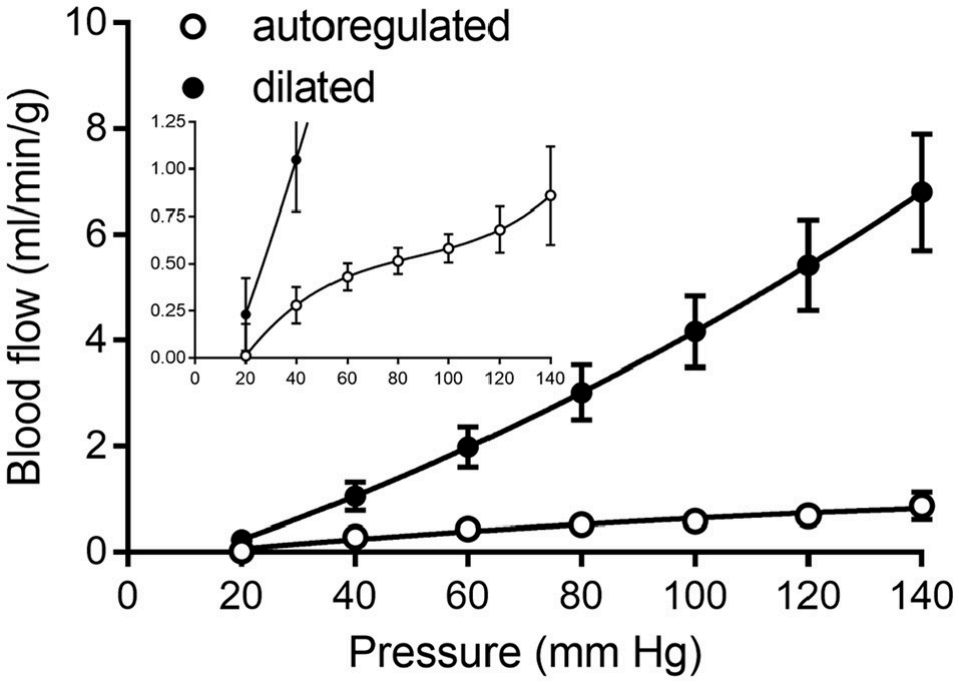
Composite of coronary pressure-flow autoregulation in swine. Open symbols represent the actively autoregulated response (Pantely et al., 1985; Johnson et al., 1988; Guth et al., 1991; McFalls et al., 1991; Schulz et al., 1991; Shnier et al., 1994; Berwick et al., 2012). Closed symbols are the pressure-flow relationship in the vasodilated coronary circulation (Pantely et al., 1985; Johnson et al., 1988; Chilian, 1991; McFalls et al., 1991; Duncker et al., 1992; Schampaert et al., 2013). The inset contains the same data, but with a magnified y-axis to appreciate the shape of the active autoregulation curve.

Curve fits of data obtained from studies in Table 2 were sampled at 20 mmHg intervals from 20 to 140 mmHg to obtain data suitable for creating composite group data with means and standard errors (Figure 8).

## Data analysis

The active myogenic parameters obtained from the 11 data sets that were fit with Equation (4) are listed in Table 3. Among the three myogenic parameters the highest certainty is in ρ_*m*_ (peak amplitude), while the least certainty resides in the parameter ϕ_*m*_ (pressure at ρ_*m*_). There is high uncertainty in fitting ϕ_*m*_, as many of the data sets do not have vessel diameters beyond pressures of 100 mmHg. During the curve fit, if φ_*m*_ and *C*_*m*_ (pressure bandwith) exceeded the maximum pressure in the data, it was truncated at that pressure.

**Table 3:** Myogenic parameters of arterioles sorted by layer and order.

**Layer**	**Order**	**ρ_m_ (μm)**	**ϕ_m_ (mmHg)**	**C_m_ (mmHg)**
Subepicardium	5	33.6 ± 8.4	91.8 ± 19.4	63.0 ± 5.1
	6	40.2 ± 18.7	96.9 ± 8.5	73.1 ± 26.7
	7	74.1	77.3	51.3
Subendocardium	6	38.9	103.0	92.6
	7	102.3	120.0	93.7

The maximum decrease in radius is ρ_m_, while the pressure at which radius decreases by ρ_m_ is ϕ_m_. The pressure bandwidth of changes in radius is C_m_.

**Figure 9:**
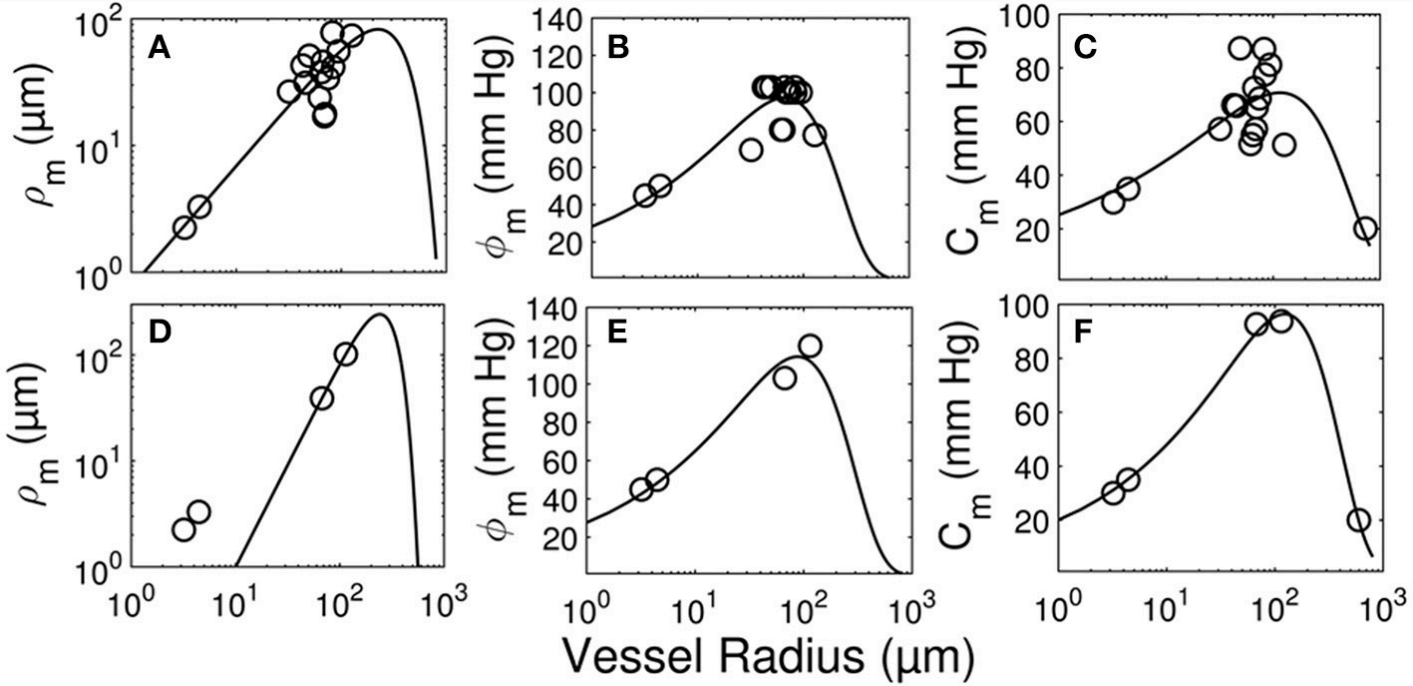
Longitudinal distribution of the myogenic parameters of coronary arterioles. Results are shown for vessels from the subepicardial (top row; **A-C**) and subendocardial (bottom row; **D-F**) layers. The left column **(A,D)** displays the maximum decrease in vessel radius, ρ_m_. The middle column **(B,E)** shows φ_m_, the transvascular pressure at which the vessel radius decreases by ρ_m_. The right column **(C,F)** displays the pressure bandwidth of changes in radius, C_m_.

The longitudinal distribution of myogenic parameters as a function of the vessel cast radius is shown in Figure 9. A Weibull fit was used to determine the distribution of parameters of vessels from the subepicardium (top panel) and subendocardium (bottom panel). The distributions of these myogenic parameters, ρ_*m*_, ϕ_*m*_, and *C*_*m*_, are model inputs to the coronary flow analysis. Among the three parameters, the myogenic amplitude, ρ_*m*_, is a sensitive indicator of the strength of the reactivity for a given vessel order. Due to the limited data in the subendocardium (only two data points), statistical analysis could not be performed for transmural differences in myogenic parameters. Further, the Weibull fit of subepicardial data have a greater uncertainty than the fit of the epicardial vessels, hence the transmural differences in myogenic parameters should be interpreted cautiously. However, the myogenic amplitude, ρ_*m*_, in vessel order 7 is 38% higher in subendocardium than subepicardium. Finding greater myogenic reactivity in subendocardial vessels of the same order contrasts with conclusions made by Kuo et al. (1988). This is not entirely surprising; however, as Sorop et al. documented very prominent myogenic reactivity in arterioles form the subendocardium (Sorop et al., 2008; Figure 7B, order 7).

**Figure 10:**
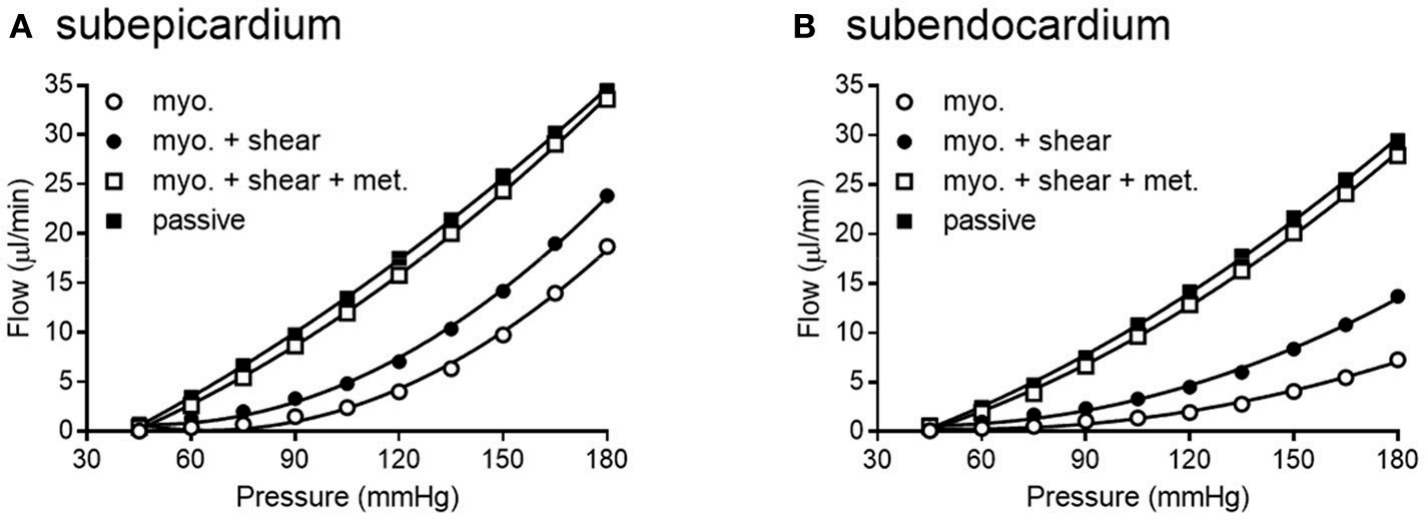
Network flow is influenced by regulatory mechanisms. **(A,B)** show flow for subepicardial and subendocardial networks, respectively. The lowest curve is flow in the presence of myogenic regulation only. The highest curve is flow in the passive state. Adding shear stress-dependent effects to the model increases flow some, but adding metabolic mechanisms brings the flow curve close to that in the passive state.

**Figure 11:**
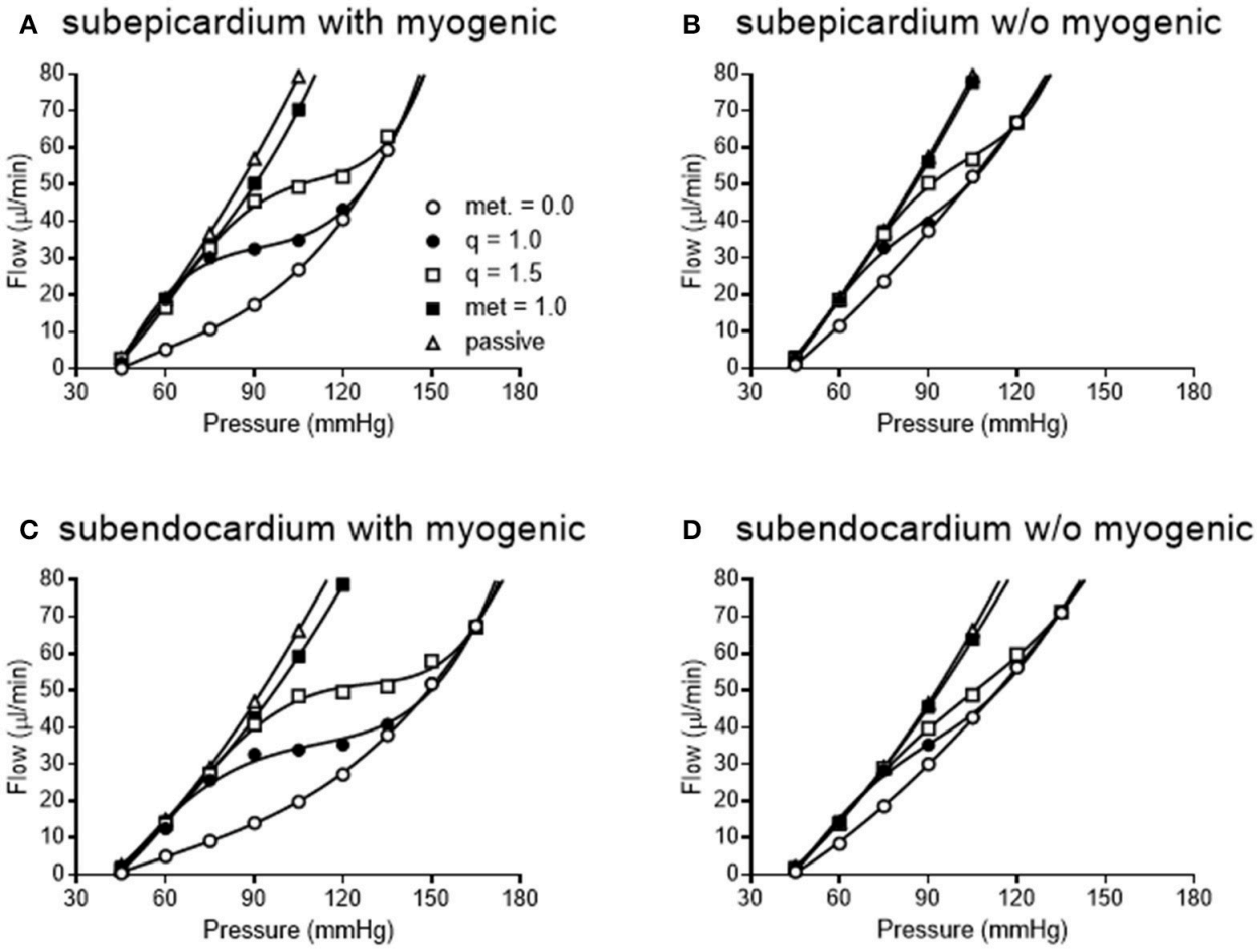
Simulated pressure-flow autoregulation curves in subepicardial **(A,B)** and subendocardial **(C,D)** subtrees. Simulations that include myogenic reactivity are in **(A,C)**. Simulations that do not consider myogenic reactivity are in **(B,D)** (vessels do have 15% tone that is independent of pressure).

We simulated coronary autoregulation with various flow control mechanisms in place (Figures 10, 11). To do so, we used our recently developed model that considers realistic anatomy and integrated passive and active determinants of flow (Namani et al., 2018). It has been proposed that physical myocardial-vessel interactions (MVI) are important in coronary flow regulation and heterogeneity (DeFily and Chilian, 1995). Our previous modeling indicates that the combined effects of cavity-induced extracellular pressure and shortening-induced intramyocyte pressure are a good reflection of intramyocardial pressure and MVI (Algranati et al., 2010). Thus, flow regulation by MVI was included in our current model. Network flow is influenced by various regulatory mechanisms and transmural location (Figure 10). Flow was lowest with myogenic regulation only, whereas flow was highest in the passive state. Adding shear stress-dependent effects increased flow over myogenic regulation alone, but adding metabolic mechanisms increased flow almost maximally (Figure 10). Flow is not autoregulated in the simulations of Figure 10. In our model, it is optimization of metabolism and the presence of myogenic responses that provides predicted flow resembling autoregulation (Figure 11). In all simulations, three control mechanism were always present: (1) metabolism (at varying levels); (2) shear; and (3) MVI. In contrast, and most importantly, simulations were run with and without myogenic reactivity, as it was our goal to determine how myogenic responses contribute to coronary pressure-flow autoregulation. When myogenic reactivity was included, the model inputs were the composite pressure-diameter relationships obtained from our analysis of the literature (Figure 7). When myogenic reactivity was removed from the simulations, it was replaced by a constant, pressure-independent tone of 15%. The autoregulation model predicts different pressure-flow patterns in the subendocardial and subepicardial layers of the heart (Figure 11; compare Figures 11A,C). Further, the autoregulation model predicts substantial changes in the pressure-flow relationship within a layer when myogenic reactivity is absent (Figure 11; compare Figures 11A,B and Figures 11C,D).

When the autoregulatory profiles of the subendocardium and subepicardium are compared, a major difference is noted. The predicted autoregulatory range in the subendocardium is greater than that in the subepicardium. Specifically, when myogenic responses are included in the simulation, the perfusion pressure range for appreciable autoregulation in the subendocardium is approximately 75–135 mmHg (Figure 11C). In contrast, in the subepicardium, when myogenic reactivity is included in the simulation, the pressure range for appreciable autoregulation is approximately 75–120 mmHg (Figure 11A). When myogenic reactivity is eliminated from the simulations, the pressure range for appreciable autoregulation considerably reduced in both layers of the myocardium. That is, autoregulatory pressure ranges in both layers are reduced to approximately 75–105 mmHg (Figures 11B,D). Thus, the myogenic response has a significant influence in regulating flow at higher perfusion pressures, as active myogenic contractions reduce flow at higher pressures and extend the autoregulatory range. The removal of myogenic responses caused the flow-perfusion curve to approach that of a passive vessel tree, demonstrating the uncoupling of myogenic regulation from flow and metabolic regulation.

**Figure 12:**
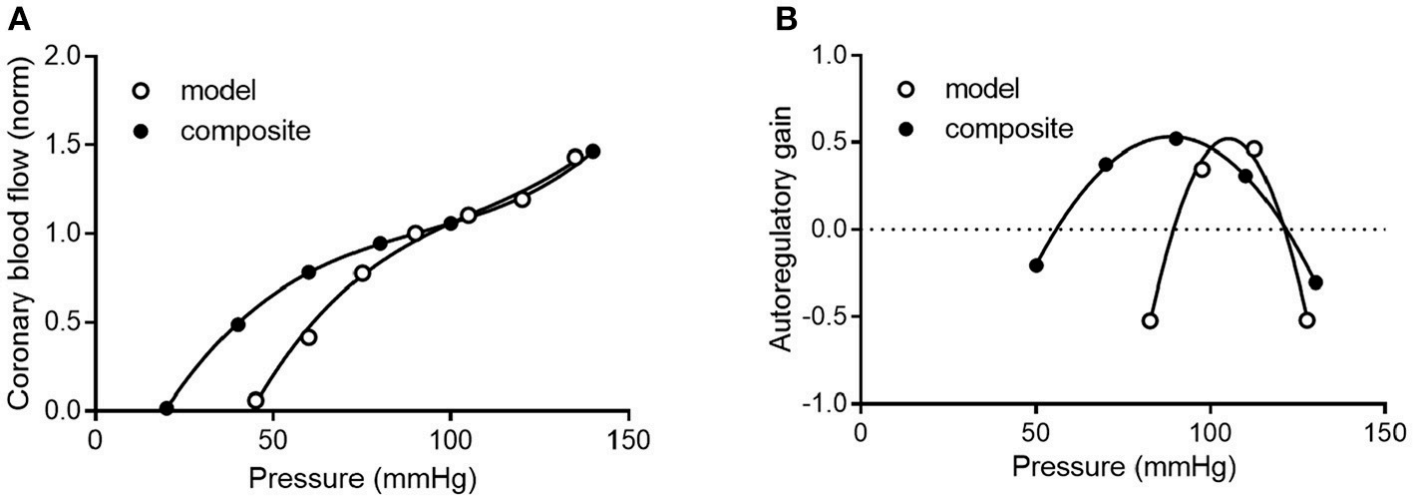
Comparison of composite and simulated coronary pressure-flow autoregulation. **(A)** shows normalized coronary pressure-flow curves for the composite data of Figure 8 and the averaged subendocardial and subepicardial data from simulations in Figures 11A,C. Data were normalized to their respective flow at 90 mmHg. **(B)** contains a comparison of autoregulatory gains calculated by Equation (8).

To determine how well the model prediction agrees with *in vivo* coronary pressure-flow autoregulation, we compared the simulation data in Figures 11A,C to the composite data of Figure 8. This analysis had two parts and is shown in Figure 12. In the first part of the comparison, both the model and composite autoregulation curves were normalized to their own respective flow values at a pressure of 90 mmHg (simulation data from the subendocardial and subepicardial layers were averaged for this comparison; Figure 12A). The composite and predicted curves are quite similar in shape, but the zero-flow pressure from the simulation is right shifted approximately 25 mmHg compared to the composite data. For the second half of the analysis, closed loop autoregulatory gain was calculated for active curves from both the model and the composite data (Figure 12B). Gain was calculated using Equation (8)

(8) $$\begin{array}{r} 1-\left[\left(\Delta \text{F}/{\text{F}}_{\text{i}}\right)/\left(\Delta \text{P}/{\text{P}}_{\text{i}}\right)\right] \end{array}$$

Where F is flow at pressure P, F_i_ and P_i_ are initial flow and pressure, ΔF is F_i_ – F, and ΔP is P_i_ – P. Positive gain values indicate active autoregulatory behavior (i.e., vasoconstriction as pressure is increased), negative gain values indicate vasodilation, while a gain of 1 is perfect autoregulation. Peak autoregulatory gains are similar (approximately 0.5 in both the simulated and actual data); however, the peak pressure for autoregulation from the simulation is right-shifted from the composite *in vivo* data by approximately 15 mmHg (Figure 12B). Further, the effective range of autoregulation predicted by the model appears to about half of that observed in the composite *in vivo* data (approximately 30 vs. 60 mmHg; Figure 12B).

## Summary, conclusions, and perspectives

Direct comparisons of coronary pressure-diameter relationships and coronary blood flow in the same species are lacking. Because data exist on both coronary myogenic reactivity (Table 1 and Figure 7) and coronary pressure-flow autoregulation in swine (Table 2 and Figure 8), we performed a meta-analysis with three parts. First, we analyzed 11 prior studies of myogenic responsiveness in swine coronary arterioles with standard criteria (converting diameters and pressures to μm and mmHg, respectively; Figure 7). Second, we used morphometry to sort the myogenic responses to diameter-defined categories (Figure 7). Third, the pressure-diameter relationships of coronary arterioles were used as an input to our recently developed integrative model of coronary blood flow regulation (Namani et al., 2018). This allowed us to compare simulated coronary pressure-flow autoregulation results to *in vivo* blood flow measurements (Figures 8, 11, 12). Our study shows that the composite myogenic reactivity of swine coronary arterioles fits with simulated pressure-flow autoregulation in the same species in a qualitative and quantitative manner. Specifically, while some differences exist (e.g., the zero-flow pressure and the pressure range of coronary autoregulation), our model simulations produce pressure-flow curves that have the same general shape and slope as what is observed from *in vivo* experiments (Figure 12A). Importantly, the magnitude of autoregulatory gain in simulations and composite data show excellent agreement (Figure 12B).

No data exist regarding myogenic responses in swine coronary arterioles of orders 1, 2, 3, or 4. These vessels all have inner diameters less than 48 μm and present methodological challenges using standard pressure myography techniques. This could possibly be remedied by using techniques developed for studying isolated nephrons (Burg perfusion; Burg et al., 1966) and successfully used to study very small arterioles (down to 12 μm) from other vascular beds (Duling et al., 1981). An advantage of Burg perfusion equipment is that can remove the necessary manual manipulation required to cannulate and secure small vessels. No data exist regarding myogenic responses in swine coronary arterioles of order 8 or larger. These vessels have thicker walls and it is difficult to image the lumen using conventional pressure myography methods.

Existing data on myogenic reactivity in swine coronary arterioles (Table 1) have been collected, analyzed, presented in standardized units, and sorted to categories based on diameter and transmural location (Figure 7). Similarly, existing data on coronary pressure-flow autoregulation in swine (Table 2) have been standardized and compiled in an orderly fashion (Figure 8). This creates data sets that are simpler to interpret and to use as inputs for models of coronary vascular regulation. Having such data available to analyze may lead to a better understanding of these important physiological phenomena. Our analysis leads us to conclude that coronary myogenic reactivity plays a role in coronary pressure-flow autoregulation in swine. In fact, it can be concluded from our modeling results that coronary myogenic responses are one essential component in producing the phenomenon of coronary pressure-flow autoregulation, as replacing myogenic contractions with pressure-independent vascular tone greatly reduced autoregulatory behavior in the simulations (Figure 11). When the composite pressure-diameter relationships of swine coronary arterioles from the literature are used as an input for our model, the predicted coronary pressure-flow autoregulation profile approaches *in vivo* observations (Figure 12). We did note some differences between the simulated and composite flow data (e.g., the zero-flow pressure of the simulation was right-shifted and the predicted pressure range of coronary autoregulation was narrower than *in vivo* observation). However, our novel model simulations produce pressure-flow curves that have the same general form and slope as what is observed *in vivo* (Figure 12A). Autoregulatory gain in simulations and the composite data show similar trends (Figure 12B). More complete and appropriate data are now available to investigate the regulation of coronary blood flow in an animal model that is highly relevant to human cardiovascular health and disease.

Existence of this data set and associated modeling tools for the coronary circulation is important because it has been known for more than 50 years that multiple mechanism (i.e., myogenic, shear stress, and metabolic mechanisms) contribute to the autoregulation of blood flow in skeletal muscle (Stainsby, 1962; Jones and Berne, 1965; Borgstrom and Gestrelius, 1987). In contrast, our understanding of the contribution and interaction of these mechanisms in the coronary circulation has lagged behind, in part, because direct evidence for myogenic contractions in coronary vessels was not available until 1988 (Kuo et al., 1988). As for modeling the interactions of multiple mechanisms in skeletal muscle, Carlson et al. showed that both the myogenic and metabolic responses are needed to overcome shear-dependent effects in skeletal muscle in order to predict autoregulation that is close to experimental observations (Carlson et al., 2008). Their regulatory scheme for skeletal muscle indicates that when arterial pressure is increased, both pressure and flow increase in the arterioles, which produces several interacting effects. First, flow increases oxygen delivery to the tissues, which attenuates the vasodilatory metabolic signal. Second, increased pressure initiates vasoconstriction by the myogenic response. Third, increased flow and pressure exert more shear stress on the vessel wall, causing vasodilation. Thus, myogenic and metabolic responses work together to oppose shear-dependent effects. Moreover, Carlson et al. concluded that the metabolic response contributed more to autoregulation of blood flow than the myogenic response (Carlson et al., 2008). An important question is whether the same conclusions hold true for coronary autoregulation. Namani et al. found that metabolic and myogenic regulation were more important inputs for modeling coronary autoregulation than were shear-dependent effects (Namani et al., 2018). Using the current data set as input for the model produces results which support the previous conclusions of Namani and colleagues for three reasons. First, network flow was highly sensitive to myogenic regulation (evident from the large difference in myogenic and passive curves in Figure 10). Second, adding shear stress-dependent effects to the model increases network flow (Figure 10). Third, network flow is highly sensitive to metabolic regulation, as full metabolic activation gives a pressure-flow relationship that is very close to the passive curve (Figure 10). Thus, in our model of the coronary circulation, while shear has significant effects, metabolism is the major vasodilatory influence. Both shear and metabolism are dilatory and work to oppose myogenic constriction. This finding highlights the need for further study into regulatory mechanisms governing the coronary circulation.

## Author contributions

GD designed the study, analyzed data, and wrote the manuscript. RN, BP, and GK analyzed data and edited the manuscript.

### Conflict of interest statement

The authors declare that the research was conducted in the absence of any commercial or financial relationships that could be construed as a potential conflict of interest.

## References

[B1] Algranati D. Kassab G. S. Lanir Y. (2010). Mechanisms of myocardium-coronary vessel interaction. Am. J. Physiol. Heart Circ. Physiol. 298, H861–H873. 10.1152/ajpheart.00925.2009 19966048 PMC2838558

[B2] Bayliss W. M. (1902). On the local reactions of the arterial wall to changes of internal pressure. J. Physiol. 28, 220–231. 10.1113/jphysiol.1902.sp000911 16992618 PMC1540533

[B3] Berwick Z. C. Moberly S. P. Kohr M. C. Morrical E. B. Kurian M. M. Dick G. M.. (2012). Contribution of voltage-dependent K^+^ and Ca^2+^ channels to coronary pressure-flow autoregulation. Basic Res. Cardiol. 107:264. 10.1007/s00395-012-0264-6 22466959 PMC3724239

[B4] Borgstrom P. Gestrelius S. (1987). Integrated myogenic and metabolic control of vascular tone in skeletal muscle during autoregulation of blood flow. Microvasc. Res. 33, 353–376. 10.1016/0026-2862(87)90028-8 3613984

[B5] Burg M. Grantham J. Abramow M. Orloff J. (1966). Preparation and study of fragments of single rabbit nephrons. Am. J. Physiol. 210, 1293–1298. 10.1152/ajplegacy.1966.210.6.1293 5923067

[B6] Carlson B. E. Arciero J. C. Secomb T. W. (2008). Theoretical model of blood flow autoregulation: roles of myogenic, shear-dependent, and metabolic responses. Am. J. Physiol. Heart Circ. Physiol. 295, H1572–H1579. 10.1152/ajpheart.00262.2008 18723769 PMC2593503

[B7] Carlson B. E. Secomb T. W. (2005). A theoretical model for the myogenic response based on the length-tension characteristics of vascular smooth muscle. Microcirculation 12, 327–338. 10.1080/10739680590934745 16020079

[B8] Chilian W. M. (1991). Microvascular pressures and resistances in the left ventricular subepicardium and subendocardium. Circ. Res. 69, 561–570. 10.1161/01.RES.69.3.561 1873859

[B9] Chilian W. M. Layne S. M. (1990). Coronary microvascular responses to reductions in perfusion pressure. Evidence for persistent arteriolar vasomotor tone during coronary hypoperfusion. Circ. Res. 66, 1227–1238. 10.1161/01.RES.66.5.1227 2335023

[B10] Cornelissen A. J. Dankelman J. VanBavel E. Spaan J. A. (2002). Balance between myogenic, flow-dependent, and metabolic flow control in coronary arterial tree: a model study. Am. J. Physiol. Heart Circ. Physiol. 282, H2224–H2237. 10.1152/ajpheart.00491.2001 12003832

[B11] Cornelissen A. J. Dankelman J. VanBavel E. Stassen H. G. Spaan J. A. (2000). Myogenic reactivity and resistance distribution in the coronary arterial tree: a model study. Am. J. Physiol. Heart Circ. Physiol. 278, H1490–H1499. 10.1152/ajpheart.2000.278.5.H1490 10775126

[B12] Davis M. J. (2012). Perspective: physiological role(s) of the vascular myogenic response. Microcirculation 19, 99–114. 10.1111/j.1549-8719.2011.00131.x 21895845

[B13] DeFily D. V. Chilian W. M. (1995). Coronary microcirculation: autoregulation and metabolic control. Basic Res. Cardiol. 90, 112–118. 10.1007/BF00789441 7646415

[B14] Dole W. P. (1987). Autoregulation of the coronary circulation. Prog. Cardiovasc. Dis. 29, 293–323. 10.1016/S0033-0620(87)80005-1 3809516

[B15] Duling B. R. Gore R. W. Dacey R. G. Jr. Damon D. N. (1981). Methods for isolation, cannulation, and *in vitro* study of single microvessels. Am. J. Physiol. 241, H108–H116. 10.1152/ajpheart.1981.241.1.H108 7195654

[B16] Duncker D. J. Bache R. J. (2008). Regulation of coronary blood flow during exercise. Physiol. Rev. 88, 1009–1086. 10.1152/physrev.00045.2006 18626066

[B17] Duncker D. J. McFalls E. O. Krams R. Verdouw P. D. (1992). Pressure-maximal coronary flow relationship in regionally stunned porcine myocardium. Am. J. Physiol. 262(6 Pt 2), H1744–H1751. 10.1152/ajpheart.1992.262.6.H1744 1621833

[B18] Feigl E. O. (1989). Coronary autoregulation. J. Hypertens. Suppl. 7, S55–S58. 2681597

[B19] Feigl E. O. Neat G. W. Huang A. H. (1990). Interrelations between coronary artery pressure, myocardial metabolism and coronary blood flow. J. Mol. Cell. Cardiol. 22, 375–390. 10.1016/0022-2828(90)91474-L 2388275

[B20] Goodwill A. G. Dick G. M. Kiel A. M. Tune J. D. (2017). Regulation of coronary blood flow. Compr. Physiol. 7, 321–382. 10.1002/cphy.c160016 28333376 PMC5966026

[B21] Guth B. D. Schulz R. Heusch G. (1991). Pressure-flow characteristics in the right and left ventricular perfusion territories of the right coronary artery in swine. Pflugers Arch. 419, 622–628. 10.1007/BF00370305 1788057

[B22] Hamza L. H. Dang Q. Lu X. Mian A. Molloi S. Kassab G. S. (2003). Effect of passive myocardium on the compliance of porcine coronary arteries. Am. J. Physiol. Heart Circ. Physiol. 285, H653–H660. 10.1152/ajpheart.00090.2003 12860566

[B23] Hill M. A. Meininger G. A. (2012). Arteriolar vascular smooth muscle cells: mechanotransducers in a complex environment. Int. J. Biochem. Cell Biol. 44, 1505–1510. 10.1016/j.biocel.2012.05.021 22677491 PMC4221252

[B24] Hoffman J. I. Spaan J. A. (1990). Pressure-flow relations in coronary circulation. Physiol. Rev. 70, 331–390. 10.1152/physrev.1990.70.2.331 2181499

[B25] Johnson P. C. (1980). The myogenic reponse, in Handbook of Physiology. The Cardiovascular System. Vascular Smooth Muscle, eds Bohr D. F. Somlyo A. P. Sparks H. V. (Bethesda, MD: American Physiological. Society), 409–442.

[B26] Johnson P. C. (1986). Autoregulation of blood flow. Circ. Res. 59, 483–495. 10.1161/01.RES.59.5.483 3542277

[B27] Johnson W. B. Malone S. A. Pantely G. A. Anselone C. G. Bristow J. D. (1988). No reflow and extent of infarction during maximal vasodilation in the porcine heart. Circulation 78, 462–472. 10.1161/01.CIR.78.2.462 2456169

[B28] Jones R. D. Berne R. M. (1965). Evidence for a metabolic mechanism in autoregulation of blood flow in skeletal muscle. Circ. Res. 17, 540–554. 10.1161/01.RES.17.6.540 5843887

[B29] Kanatsuka H. Lamping K. G. Eastham C. L. Marcus M. L. (1990). Heterogeneous changes in epimyocardial microvascular size during graded coronary stenosis. Evidence of the microvascular site for autoregulation. Circ. Res. 66, 389–396. 10.1161/01.RES.66.2.389 2297810

[B30] Kassab G. S. Rider C. A. Tang N. J. Fung Y. C. (1993). Morphometry of pig coronary arterial trees. Am. J. Physiol. 265(1 Pt 2), H350–H365. 10.1152/ajpheart.1993.265.1.H350 8342652

[B31] Kuo L. Chilian W. M. Davis M. J. (1990a). Coronary arteriolar myogenic response is independent of endothelium. Circ. Res. 66, 860–866. 10.1161/01.RES.66.3.860 2306810

[B32] Kuo L. Chilian W. M. Davis M. J. (1991). Interaction of pressure- and flow-induced responses in porcine coronary resistance vessels. Am. J. Physiol. 261(6 Pt 2), H1706–H1715. 10.1152/ajpheart.1991.261.6.H1706 1750529

[B33] Kuo L. Davis M. J. Chilian W. M. (1988). Myogenic activity in isolated subepicardial and subendocardial coronary arterioles. Am. J. Physiol. 255(6 Pt 2), H1558–H1562. 10.1152/ajpheart.1988.255.6.H1558 2462367

[B34] Kuo L. Davis M. J. Chilian W. M. (1990b). Endothelium-dependent, flow-induced dilation of isolated coronary arterioles. Am. J. Physiol. 259(4 Pt 2), H1063–H1070. 10.1152/ajpheart.1990.259.4.H1063 2221113

[B35] Lelovas P. P. Kostomitsopoulos N. G. Xanthos T. T. (2014). A comparative anatomic and physiologic overview of the porcine heart. J. Am. Assoc. Lab. Anim. Sci. 53, 432–438. 25255064 PMC4181683

[B36] Liao J. C. Kuo L. (1997). Interaction between adenosine and flow-induced dilation in coronary microvascular network. Am. J. Physiol. 272(4 Pt 2), H1571–H1581. 10.1152/ajpheart.1997.272.4.H1571 9139938

[B37] McFalls E. O. Duncker D. J. Sassen L. M. Gho B. C. Verdouw P. D. (1991). Effect of antiischemic therapy on coronary flow reserve and the pressure-maximal coronary flow relationship in anesthetized swine. J. Cardiovasc. Pharmacol. 18, 827–836. 10.1097/00005344-199112000-00007 1725894

[B38] Merkus D. Vergroesen I. Hiramatsu O. Tachibana H. Nakamoto H. Toyota E.. (2001). Stenosis differentially affects subendocardial and subepicardial arterioles *in vivo*. Am. J. Physiol. Heart Circ. Physiol. 280, H1674–H1682. 10.1152/ajpheart.2001.280.4.H1674 11247779

[B39] Mosher P. Ross J. Jr. McFate P. A. Shaw R. F. (1964). Control of coronary blood flow by an autoregulatory mechanism. Circ. Res. 14, 250–259. 10.1161/01.RES.14.3.250 14133952

[B40] Muller J. M. Myers P. R. Laughlin M. H. (1993). Exercise training alters myogenic responses in porcine coronary resistance arteries. J. Appl. Physiol. 75, 2677–2682. 10.1152/jappl.1993.75.6.2677 8125889

[B41] Nakayama K. Osol G. Halpern W. (1988). Reactivity of isolated porcine coronary resistance arteries to cholinergic and adrenergic drugs and transmural pressure changes. Circ. Res. 62, 741–748. 10.1161/01.RES.62.4.741 3349575

[B42] Namani R. Kassab G. S. Lanir Y. (2018). Integrative model of coronary flow in anatomically based vasculature under myogenic, shear, and metabolic regulation. J. Gen. Physiol. 150, 145–168. 10.1085/jgp.201711795 29196421 PMC5749109

[B43] Pantely G. A. Bristow J. D. Swenson L. J. Ladley H. D. Johnson W. B. Anselone C. G. (1985). Incomplete coronary vasodilation during myocardial ischemia in swine. Am. J. Physiol. 249(3 Pt 2), H638–H647. 10.1152/ajpheart.1985.249.3.H638 4037109

[B44] Rajagopalan S. Dube S. Canty J. M. Jr. (1995). Regulation of coronary diameter by myogenic mechanisms in arterial microvessels greater than 100 microns in diameter. Am. J. Physiol. 268(2 Pt 2), H788–H793. 10.1152/ajpheart.1995.268.2.H788 7864206

[B45] Schampaert S. van 't Veer M. Rutten M. C. van Tuijl S. de Hart J. van de Vosse F. N.. (2013). Autoregulation of coronary blood flow in the isolated beating pig heart. Artif. Organs 37, 724–730. 10.1111/aor.12065 23489228

[B46] Schulz R. Guth B. D. Heusch G. (1991). No effect of coronary perfusion on regional myocardial function within the autoregulatory range in pigs. Evidence against the Gregg phenomenon. Circulation 83, 1390–1403. 10.1161/01.CIR.83.4.1390 2013156

[B47] Shnier C. B. Cason B. A. Horton A. F. Hickey R. F. (1994). Coronary blood flow autoregulation and flow heterogeneity in the stunned heart. Jpn. Heart J. 35, 654–660. 10.1536/ihj.35.645 7830329

[B48] Sorop O. Merkus D. de Beer V. J. Houweling B. Pistea A. McFalls E. O.. (2008). Functional and structural adaptations of coronary microvessels distal to a chronic coronary artery stenosis. Circ. Res. 102, 795–803. 10.1161/CIRCRESAHA.108.172528 18292598

[B49] Stainsby W. N. (1962). Autoregulation of blood flow in skeletal muscle during increased metabolic activity. Am. J. Physiol. 202, 273–276. 10.1152/ajplegacy.1962.202.2.273 13916114

[B50] Suzuki Y. Yeung A. C. Ikeno F. (2011). The representative porcine model for human cardiovascular disease. J. Biomed. Biotechnol. 2011:195483. 10.1155/2011/195483 21253493 PMC3022214

[B51] Tofukuji M. Stamler A. Li J. Franklin A. Wang S. Y. Hariawala M. D.. (1997a). Effects of magnesium cardioplegia on regulation of the porcine coronary circulation. J. Surg. Res. 69, 233–239. 10.1006/jsre.1997.5003 9224388

[B52] Tofukuji M. Stamler A. Li J. Hariawala M. D. Franklin A. Sellke F. W. (1997b). Comparative effects of continuous warm blood and intermittent cold blood cardioplegia on coronary reactivity. Ann. Thorac. Surg. 64, 1360–1367. 10.1016/S0003-4975(97)00990-9 9386705

[B53] Tune J. D. (2014). Coronary circulation, in Colloquium Series on Integrated Systems Physiology: From Molecule to Function to Disease, Vol 6 (Morgan & Claypool Life Sciences), 1–189.

[B54] Ueeda M. Silvia S. K. Olsson R. A. (1992). Nitric oxide modulates coronary autoregulation in the guinea pig. Circ. Res. 70, 1296–1303. 10.1161/01.RES.70.6.1296 1374299

[B55] Wang S. Y. Friedman M. Franklin A. Sellke F. W. (1995). Myogenic reactivity of coronary resistance arteries after cardiopulmonary bypass and hyperkalemic cardioplegia. Circulation 92, 1590–1596. 10.1161/01.CIR.92.6.1590 7664445

[B56] Wang S. Y. Stamler A. Tofukuji M. Deuson T. E. Sellke F. W. (1997). Effects of blood and crystalloid cardioplegia on adrenergic and myogenic vascular mechanisms. Ann. Thorac. Surg. 63, 41–49. 10.1016/S0003-4975(96)00644-3 8993239

[B57] Young J. M. Choy J. S. Kassab G. S. Lanir Y. (2012). Slackness between vessel and myocardium is necessary for coronary flow reserve. Am. J. Physiol. Heart Circ. Physiol. 302, H2230–H2242. 10.1152/ajpheart.01184.2011 22408024 PMC3378297

